# A Study on the Recycling of Sodium Citrate (Na_3_Cit) Waste Solution via Bipolar Membrane Electrodialysis

**DOI:** 10.3390/membranes16090284

**Published:** 2026-08-27

**Authors:** Young-Jae Lee, Min-Hyuk Seo, Jae-Hyuk Chang, Jun-Hee Kim, Jae-Woo Ahn

**Affiliations:** Department of Advanced Materials Science and Engineering, Daejin University, Pocheon 11159, Republic of Korea; youngjae1425@naver.com (Y.-J.L.); smhyuk1224@naver.com (M.-H.S.); ab225026@daejin.ac.kr (J.-H.C.); ab225025@daejin.ac.kr (J.-H.K.)

**Keywords:** bipolar membrane electrodialysis, citric acid, two-compartment, NaOH, constant current

## Abstract

Citric acid-based leaching is gaining attention as an environmentally friendly alternative to conventional sulfuric acid-based processes for recycling spent lithium-ion batteries (LIBs), but it generates sodium citrate (Na_3_Cit)-rich wastewater that is difficult to treat using conventional technologies. Bipolar membrane (BM) electrodialysis (BMED), particularly a two-compartment BM/cation-exchange membrane (CEM) configuration, offers a simple and energy-efficient solution for simultaneously recovering acids and bases from such wastewater without external reagents, enabling a closed-loop resource-circulation approach that remains largely unexplored for this specific waste stream. This system was applied to treat Na_3_Cit wastewater generated from citric acid-based spent LIB leaching, and the recovery feasibility and process performance of citric acid and NaOH were evaluated. The effects of feed concentration, current density, initial base concentration, and initial base volume on NaOH recovery, current efficiency, and energy consumption were investigated. Under the optimal conditions (1.00 M Na_3_Cit, 300 A/m^2^, 0.1 M NaOH, 1.25 L), a NaOH recovery of 93.93%, current efficiency of 92.55%, and energy consumption of 0.65 kWh/kg were achieved. This study demonstrates that Na_3_Cit wastewater can be treated via BMED without external reagents, yielding high-purity NaOH, whereas the recovered acid stream contains residual unreacted Na_3_Cit, and its direct reuse in the leaching process requires further verification. These findings provide a fundamental basis for developing BMED-based resource-circular processes.

## 1. Introduction

Citric acid (C_6_H_8_O_7_) is a representative organic acid widely used as a key material in various industries. It serves as an acidity regulator and a pH buffering agent in the food and beverage industry, a stabilizer in the pharmaceutical and cosmetic fields, and a chelating agent in metal cleaning and electroplating processes [1,2]. Owing to its high biodegradability, lower corrosivity compared with inorganic acids, and excellent ability to form complexes with multivalent metal ions, its applications are continuously expanding in an industrial environment with increasingly stringent environmental regulations [3]. The global citric acid market, driven by this expansion, is approximately 3 million tons as of 2024 and is projected to grow at a compound annual growth rate (CAGR) of approximately 2.5% from 2025 to 2033, reaching approximately 3.7 million tons by 2033 [4].

One notable industrial application of citric acid is its role as a leaching agent in the recycling of spent lithium-ion batteries (LIBs). With the rapid growth in demand for electric vehicles, portable electronic devices, and energy storage systems (ESSs), the amount of spent LIBs is rapidly increasing. The recovery of valuable metals such as Li, Co, Ni, and Mn from spent LIBs is strategically important for resource circulation and supply chain stability. Accordingly, there exists a growing demand for safer and more environmentally friendly leaching agents to replace conventional strong acid-based ones. The citric acid-based leaching process is gaining attention as an environmentally friendly alternative to the conventional sulfuric acid (H_2_SO_4_)-based process because it is less corrosive, minimizes sulfate byproduct generation, and produces highly biodegradable wastewater [5,6]. Recent studies show that citric acid achieves excellent leaching efficiency of 85–96% for Li, Co, Ni, and Mn even under high-concentration, high solid-to-liquid ratio (S/L) conditions [7]. Moreover, using a citric acid-based EDTA-H_2_O_2_ buffer system can improve metal recovery efficiency by increasing the stability of Co·Ni·Mn complexes [8]. Overall, the citric acid-based leaching process is a technology with high potential for application in spent LIB recycling because of its high metal recovery efficiency and environmental friendliness. However, a key challenge remains in the commercialization of the citric acid-based leaching process. After the leaching-metal separation step, the final process residual solution contains sodium citrate (Na_3_Cit), which accumulates to high concentrations and generates wastewater containing residual metal–citrate complexes, high concentrations of Na^+^, and other substances [9]. This wastewater considerably increases dissolved organic carbon (TOC) and chemical oxygen demand (COD), increasing the burden on downstream processes [10]; therefore, achieving both organic matter removal and metal separation via simple neutralization and dilution is challenging. Technologies under review for this treatment include nanofiltration (NF), reverse osmosis (RO), advanced oxidation processes (AOPs), and electrochemical advanced oxidation processes (EAOPs). However, for NF and RO, membrane fouling and scaling readily occur owing to high concentrations of organic salts and metal complexes, and the unresolved issue of concentrate treatment hinders their practical use for resource recovery [11,12]. For AOPs and EAOPs, degradation efficiency is low, and energy consumption is high because of the high structural stability of citrate complexes, making economic feasibility difficult to achieve [13,14]. Thus, existing treatment technologies have fundamental limitations in simultaneously achieving organic matter removal and resource recovery. Therefore, developing a new resource-circulating treatment technology is necessary to regenerate organic acids from citrate wastewater and recycle process water.

**Figure 1:**
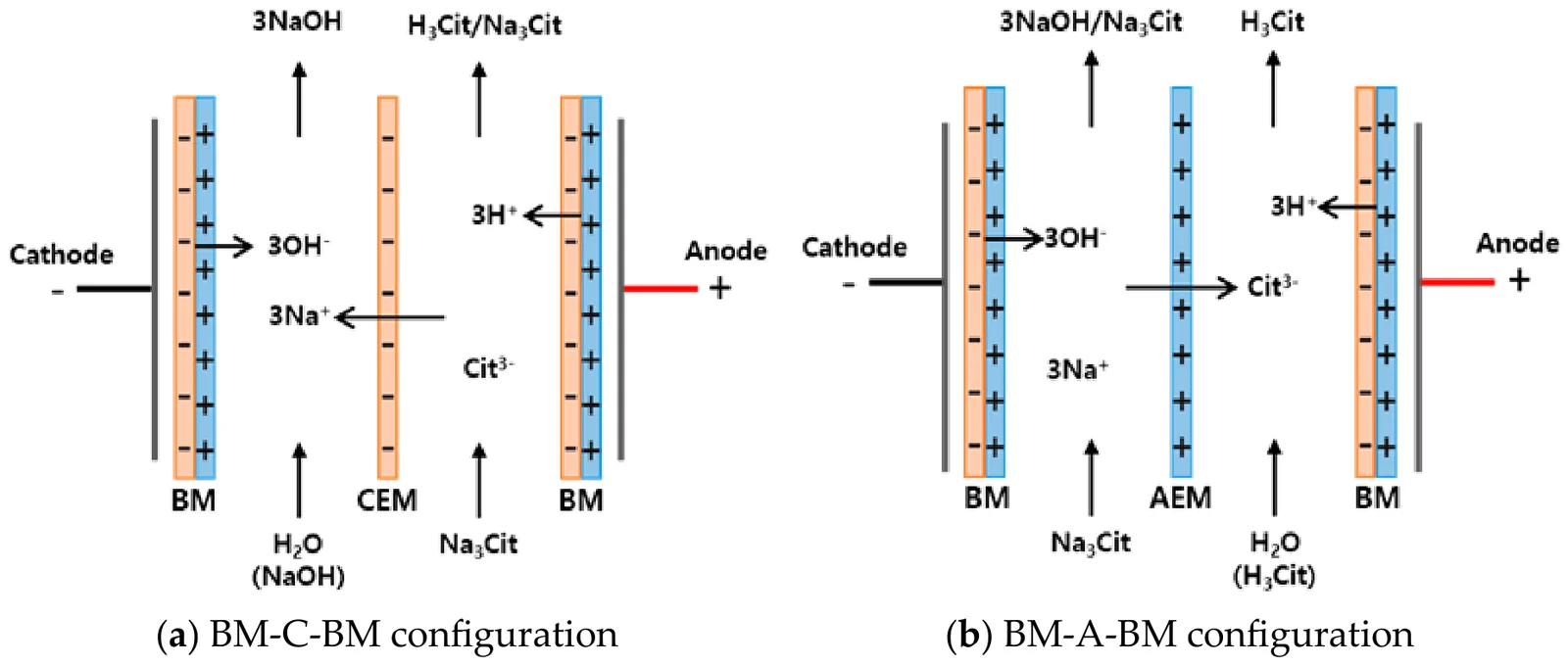
Two-compartment BMED system.

Bipolar membrane (BM) electrodialysis (BMED) is a promising electrochemical technology to address these challenges. BMED can produce and recover acids and bases from organic or inorganic salt solutions without adding external acid or base reagents by combining the selective ion transport of ion exchange membranes (cation exchange membrane [CEM] and anion exchange membrane [AEM]) with the water-splitting reaction inside a bipolar membrane (BM) [15,16]. Depending on cell configuration, BMED systems are classified into a three-compartment system (BM/CEM/AEM) or a two-compartment system (BM/AEM or BM/CEM). A three-compartment system separates acid and base recovery chambers, allowing the acid and base to be recovered in pure form. However, this system increases stack resistance and energy consumption due to the high number of membranes. In contrast, a two-compartment system, as shown in Figure 1, recovers an acid or a base mixed with salt but offers a simpler membrane configuration, resulting in lower stack resistance and higher energy efficiency [17]. In fact, BMED systems have been applied to various organic acid salt wastewaters, such as those containing citric acid and lactic acid, and their feasibility for acid and base recovery has been demonstrated [18,19,20]. In particular, a two-compartment system with a BM/CEM configuration enables a reaction pathway in which H^+^ generated at the BM protonates Cit^3−^ in the feed solution to regenerate citric acid (H_3_Cit), while Na^+^ is transported through the CEM to the base recovery chamber to produce NaOH. Therefore, this system is suitable for treating Na_3_Cit wastewater.

This study aimed to evaluate the recovery potential of citric acid (H_3_Cit) and sodium hydroxide (NaOH), as well as process performance, by applying a two-compartment (BM/CEM) BMED system to Na_3_Cit wastewater generated from a citric acid-based leaching process. The effects of the main operating parameters, namely, feed solution concentration, current density, initial base concentration, and initial base volume, on NaOH recovery, current efficiency, and energy consumption were systematically analyzed. The study aimed to determine the optimal operating conditions for efficiently recovering citric acid and NaOH from Na_3_Cit wastewater and to provide foundational data for developing a BMED-based resource circulation process.

## 2. Materials and Methods

### 2.1. Experimental Apparatus

**Figure 2:**
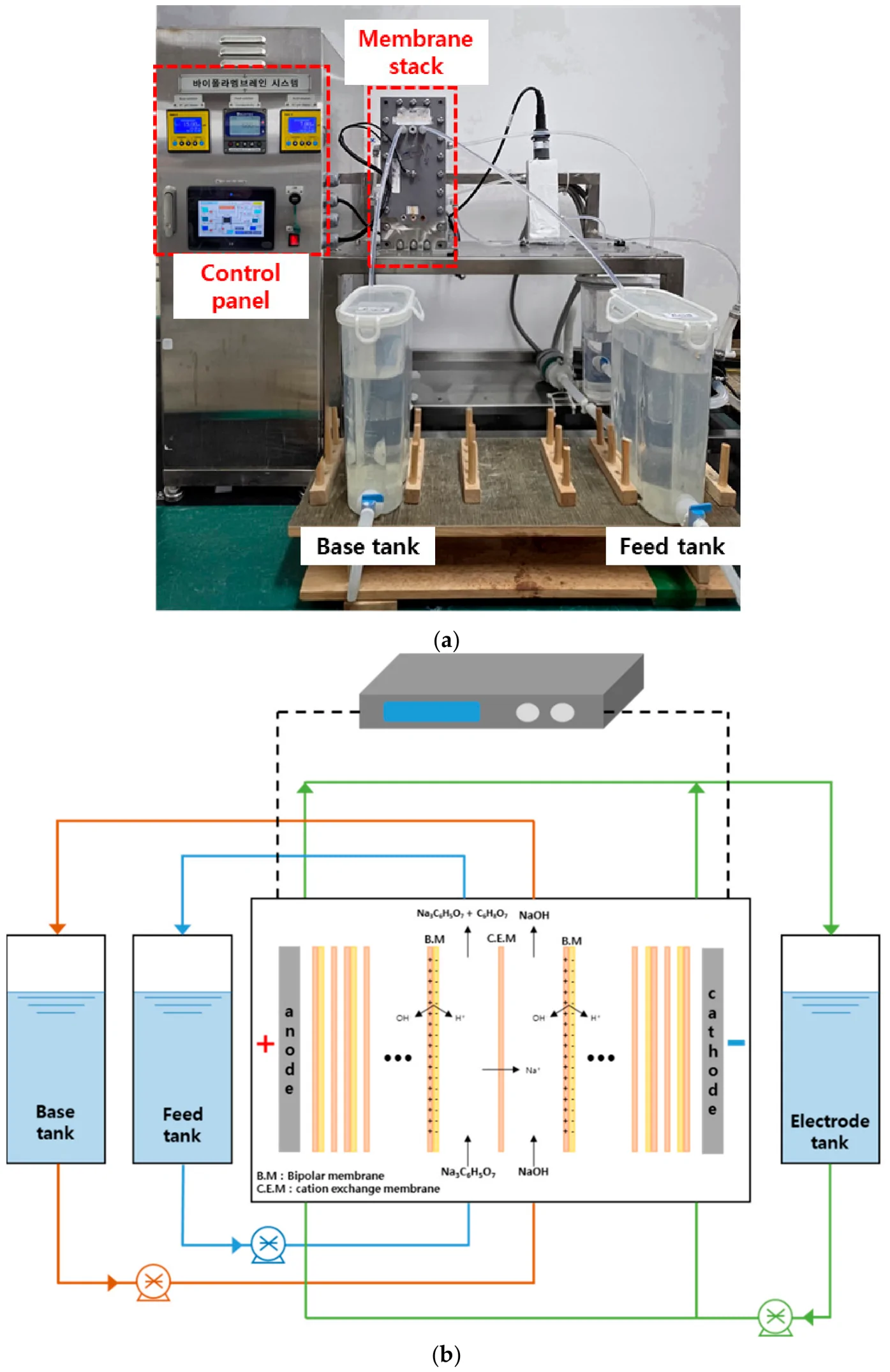
(**a**) Photograph and (**b**) schematic diagram of a two-compartment BMED system.

In this study, a lab-scale two-compartment BM/CEM-type device was fabricated in-house and used. A photograph and a schematic of the device are shown in Figure 2, and its main specifications are presented in Table 1. The apparatus comprises a base chamber, a feed chamber, an electrode chamber, a membrane stack, a pH meter, an electrical conductivity meter, a pump, and a rectifier. Platinum-coated titanium plates were used as the electrodes. Approximately 2 L of 0.1 M NaOH was used as the electrode solution, which was circulated between the electrode and the outermost membrane of the stack via a dedicated tank. All flow paths between the solution tank, pump, and stack are constructed using PFA tubing with an inner diameter of 4 mm, and the solutions were circulated at an average flow rate of 950 mL/min using a circulation pump (MD-10, Iwaki Co., Ltd., Tokyo, Japan). To suppress temperature increases due to pump operation and membrane resistance, the pump and solution tank are designed with an externally exposed structure to allow natural air cooling. In addition, an air cooler was separately installed, and the feed solution temperature was monitored in real time to maintain it at 30 °C. The device has a built-in PC that enables the setting of the operating modes (constant voltage and constant current), termination conditions (electrical conductivity, voltage, and current), and individual control of pumps while recording the electrical conductivity of the feed solution, the pH of the base solution, and voltage and current data in real time. The two-compartment membrane stack uses Neosepta series BMs and CEMs from ASTOM, Tokyo, Japan. The stack comprises 11 BMs and 10 CEMs, with an effective membrane area of 0.055 m^2^. The detailed specifications of the membranes are shown in Table 2.

### 2.2. Experimental Methods

The citric acid-based recycling process for spent LIBs has not yet been commercialized and remains under development. Process simulations based on previously reported studies identified Na_3_Cit as the major component of the expected wastewater. Since actual industrial wastewater does not yet exist, a synthetic Na_3_Cit solution reflecting this simulated composition was used as a model wastewater to evaluate resource recovery via bipolar membrane electrodialysis (BMED). The experiment was conducted using the aforementioned BMED apparatus. The same membrane stack was used throughout all experiments in this study; after each experimental run, the membrane stack was thoroughly washed to remove residual solutes and prevent membrane fouling before proceeding to the subsequent experiment. Anhydrous trisodium citrate (Na_3_Cit, C_6_H_5_Na_3_O_7_, molecular weight 258.07 g/mol) was used to prepare the feed solution. Depending on the conditions, 1.5 L of a 0.30–1.50 M Na_3_Cit solution was used as the feed solution, and 0.1 M NaOH was used in the base chamber as the baseline condition. The volume ratio between the feed and base solutions is denoted as F/B (L/L) throughout this study. To evaluate the effects of the initial base concentration and volume, the initial charge concentration and charge volume were varied in the ranges of 0.1–1.0 M and 0.5–2.0 L, respectively. All experiments were conducted under constant-current conditions, and the experiment termination condition was set as the point at which the system voltage reached 25 V, the maximum safe operating voltage recommended by the membrane manufacturer. During operation, the voltage, current, and electrical conductivity of the feed solution were automatically recorded at 1 min intervals. The volume change in each solution compartment before and after the experiment was determined gravimetrically and used to calculate water migration. Samples were collected from the feed solution and the base solution at regular intervals to monitor acid–base generation and ion transport. For analyzing the feed solution, the degree of conversion to citric acid was evaluated by measuring the pH of the collected samples. Because citric acid (H_3_Cit) is predominantly present in its fully protonated form at a pH of 2.3 or lower, this pH value was set as the criterion indicating that the citrate species were predominantly converted to H_3_Cit. In addition, the total acidity of the feed solution was quantified via neutralization titration with 0.1 M NaOH using phenolphthalein as the indicator. In the base solution analysis, the OH^−^ concentration in the base recovery chamber was quantified via acid–base titration with 0.1 M H_2_SO_4_ using methyl orange as the indicator. Moreover, the NaOH production amount and recovery were calculated from the results. The Na^+^ concentration in each solution was precisely quantified via inductively coupled plasma–optical emission spectroscopy (ICP-OES) (Agilent Technologies, Santa Clara, CA, USA) and was used along with the titration results for ion resin analysis and data reliability verification. In addition, the residual Na^+^ concentration in the feed chamber at the end of the experiment was analyzed via ICP-OES to determine the amount of unreacted Na_3_Cit remaining in the recovered acid. Based on the results, the base recovery, generated acid concentration, current efficiency, and energy consumption were calculated.

The experimental data analysis included calculations of base recovery (%), water migration ($W_{mig}(\%)$), current efficiency (C.E, %), and energy consumption (kWh/kg, E). The base recovery (recovery, %) was calculated using Equation (1):

(1) $$Recovery\;\left(\%\right)=\frac{m_{f}}{m_{i}}\times 100$$

Here, $m_{f}$ is the moles of Na^+^ transferred to the base recovery chamber and $m_{i}$ is the moles of Na^+^ in the initial feed solution.

The water migration ($W_{mig}(\%)$) was calculated using Equation (2):

(2) $$W_{mig.}\left(\%\right)=\frac{V_{f}-V_{i}}{V_{i}}\times 100$$

Here, $v_{i}$ represents the initial volume of the solution and $v_{f}$ represents the volume of the solution after the experiment.

Current efficiency (CE, %) was calculated using Equation (3):

(3) $$CE\;\left(\%\right)=\frac{m_{actual}}{m_{theoretical}}\times 100$$

Here, $m_{theoretical}$ represents the theoretical yield, and $m_{actual}$ represents the actual yield, both normalized per single membrane pair in the stack.

The $m_{theoretical}$ was calculated using Equation (4):

(4) $$m_{theoretical}=\frac{I\times t\times M}{z\times F}$$

Here, I is the applied current (A), t is the operation time (s), M is the atomic weight of Na (22.99 g/mol), z is the valence of Na^+^ (z=1), and F is Faraday’s constant (96,485 C/mol).

The energy consumption ($E_{Weight}$) was calculated based on the Na_3_Cit throughput (kg) using Equation (4):

(5) $$E_{weight}=\frac{I\times \int_{0}^{t}Udt}{W}$$

Here, U is the average voltage (V) applied during the process, I is the applied current (A), t is the duration (hr), and W is the initial input weight (kg) of Na_3_Cit. This basis was selected because the energy consumption is intended to represent the energy required to treat a target amount of Na_3_Cit waste (e.g., for annual treatment capacity planning), rather than the energy required to obtain a given amount of recovered product.

## 3. Results and Discussion

### 3.1. Effect of Initial Feed Solution (Na_3_Cit) Concentration

**Figure 3:**
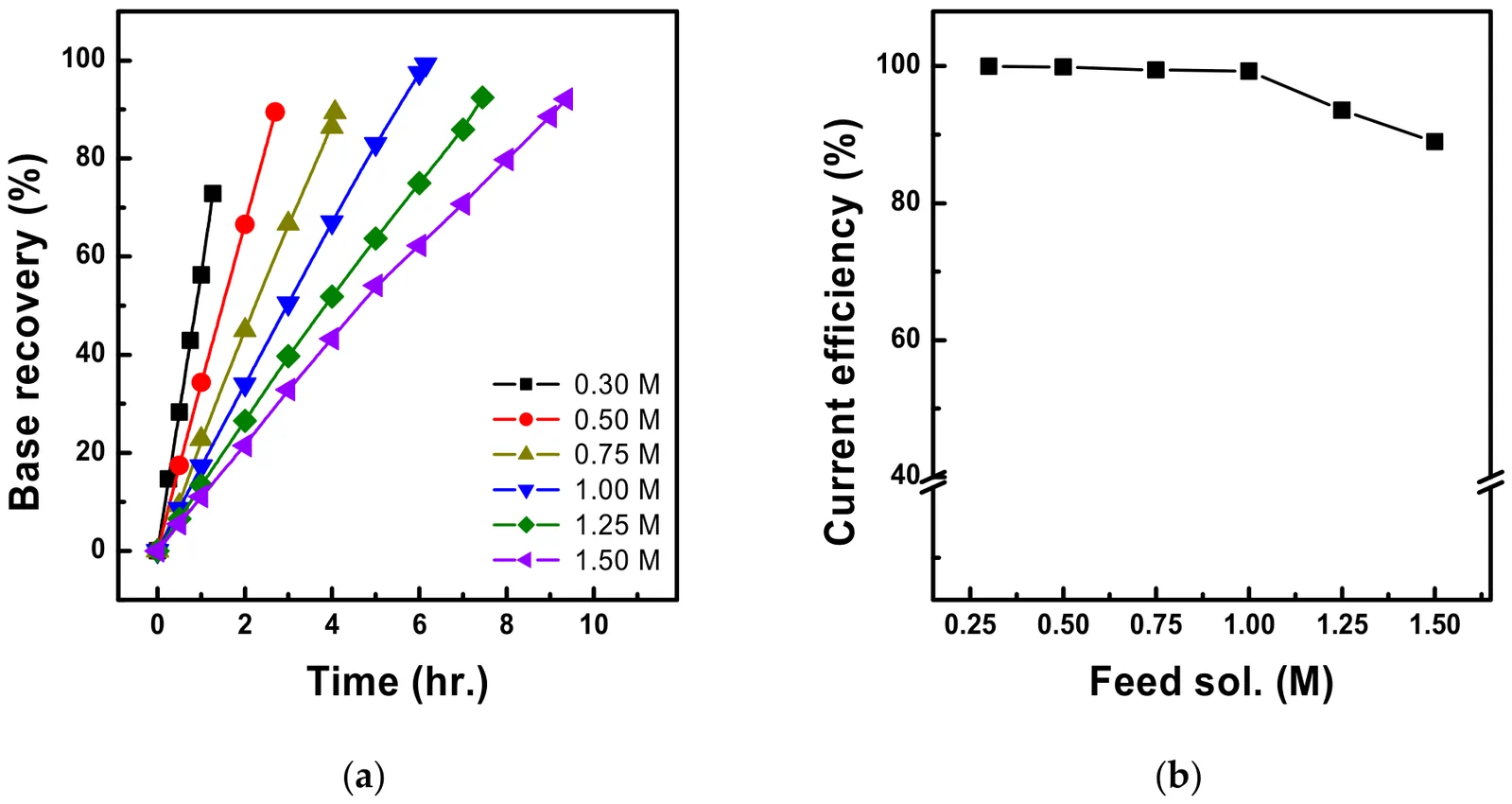
Effect of the initial concentration of the feed solution (M) on (**a**) NaOH recovery and (**b**) current efficiency at 360 A/m^2^ (0.1 M NaOH, F/B = 1.5/1.5(L), 30 °C).

To evaluate the effect of the initial feed solution concentration on the performance of the two-compartment BMED process, experiments were conducted at a current density of 360 A/m^2^ by adjusting the feed solution concentration in the range of 0.30–1.50 M. The base recovery chamber was charged with 0.1 M NaOH; moreover, 1.5 L of the feed solution and 1.5 L of the initial base solution were used. The experiment was terminated when the system voltage reached 25 V. Changes in the base recovery and current efficiency are shown in Figure 3. As shown in Figure 3a, the base recovery varied considerably depending on the feed solution concentration. At the low concentration of 0.30 M, the recovery was the lowest at 72.83%. This was likely because (a) concentration polarization occurred rapidly on the front surface of the cation-exchange membrane due to low ionic strength, and (b) the supply rate of Na^+^ could not keep up with the ion transfer rate required by the current. This behavior is consistent with the limiting current behavior reported for ED/BMED systems [21] and requires a high voltage due to decreased solution conductivity. In fact, under the 0.30-M condition, the system voltage reached 25 V after only 76 min of operation, resulting in early termination. The pH at the end of the experiment was 3.24, indicating insufficient citric acid conversion. Therefore, concentrations of 0.50 M or less are unsuitable for this process. In contrast, the 1.00-M condition showed the highest base recovery of 99.20%, and the concentration of recovered NaOH was 2.52 M. Under high-concentration conditions exceeding 1.00 M, the recovery decreased to 92.45% at 1.25 M and 92.10% at 1.50 M. This result may be attributable to the combined effect of increased mass transfer resistance due to higher solution viscosity and the back diffusion of OH^−^ ions caused by the high accumulation of NaOH. As shown in Figure 3b, the current efficiency remained high at approximately 99% in the 0.30–1.00 M range but decreased to 93.54% at 1.25 M and 88.94% at 1.50 M, respectively. The decrease in current efficiency at high concentrations is attributable to a reduction in effective ion transport current caused by increased co-ion leakage and OH^−^ back-diffusion due to the weakening of the Donnan potential resulting from a higher counter-ion concentration within the CEM, as reported in previous studies [22].

Table 3 shows the overall experimental results, including operating time, water migration, and energy consumption, as a function of feed solution concentration. As the feed solution concentration increased, operating time considerably increased from 76 min at 0.30 M to 562 min at 1.50 M, and the water migration to the base recovery chamber also increased from 5.66% to 28.96%. This is attributable to the difference in the cumulative amount of water transport with increased operating time, rather than the effect of concentration itself. The concentration of citric acid (H_3_Cit) generated in the feed solution chamber increased linearly with the feed solution concentration, reaching a maximum of 2.02 M at 1.50 M. However, because citric acid is recovered as a complex salt mixed with residual Na_3_Cit in the feed solution chamber, further research is required for separation and recovery of pure H_3_Cit. The energy consumption showed a minimum value of 0.59 kWh/kg at 0.75 M and was relatively high at 0.71 kWh/kg under the 0.30-M and 1.50-M conditions. This result may be attributable to the combined effects of increased electrolyte resistance at low concentrations, inefficient power consumption due to back-diffusion, and inhibition of ion transport at high concentrations.

### 3.2. Current Density Effect

To evaluate the effect of current density on the performance of the two-compartment BMED process, an experiment was conducted by adjusting the current density in the range of 250–450 A/m^2^. The feed solution chamber and the base recovery chamber were charged with 1.5 L of 1.00 M Na_3_Cit and 0.1 M NaOH solutions, respectively. However, at 450 A/m^2^ or higher, the cell voltage exceeded 30 V at the start of the experiment, making it impossible to maintain a constant current due to the rectifier’s capacity limit. Therefore, the upper limit of the current density was set to 450 A/m^2^.

The key process performance indicators according to changes in current density are presented in Table 4. The operating time was longest at 525 min under the lowest current density of 250 A/m^2^ and gradually decreased as the current density increased to 248 min at 450 A/m^2^, a reduction of approximately 52.8% compared to the 250 A/m^2^ condition. The water transfer rate to the base recovery chamber showed a slight overall decrease with increasing current density, reaching its lowest value of 20.10% at 450 A/m^2^. This result is likely attributable to the reduction in cumulative water migration due to the shortened operating time, which outweighs the electro-osmotic drag-induced water transport under high-current conditions.

**Figure 4:**
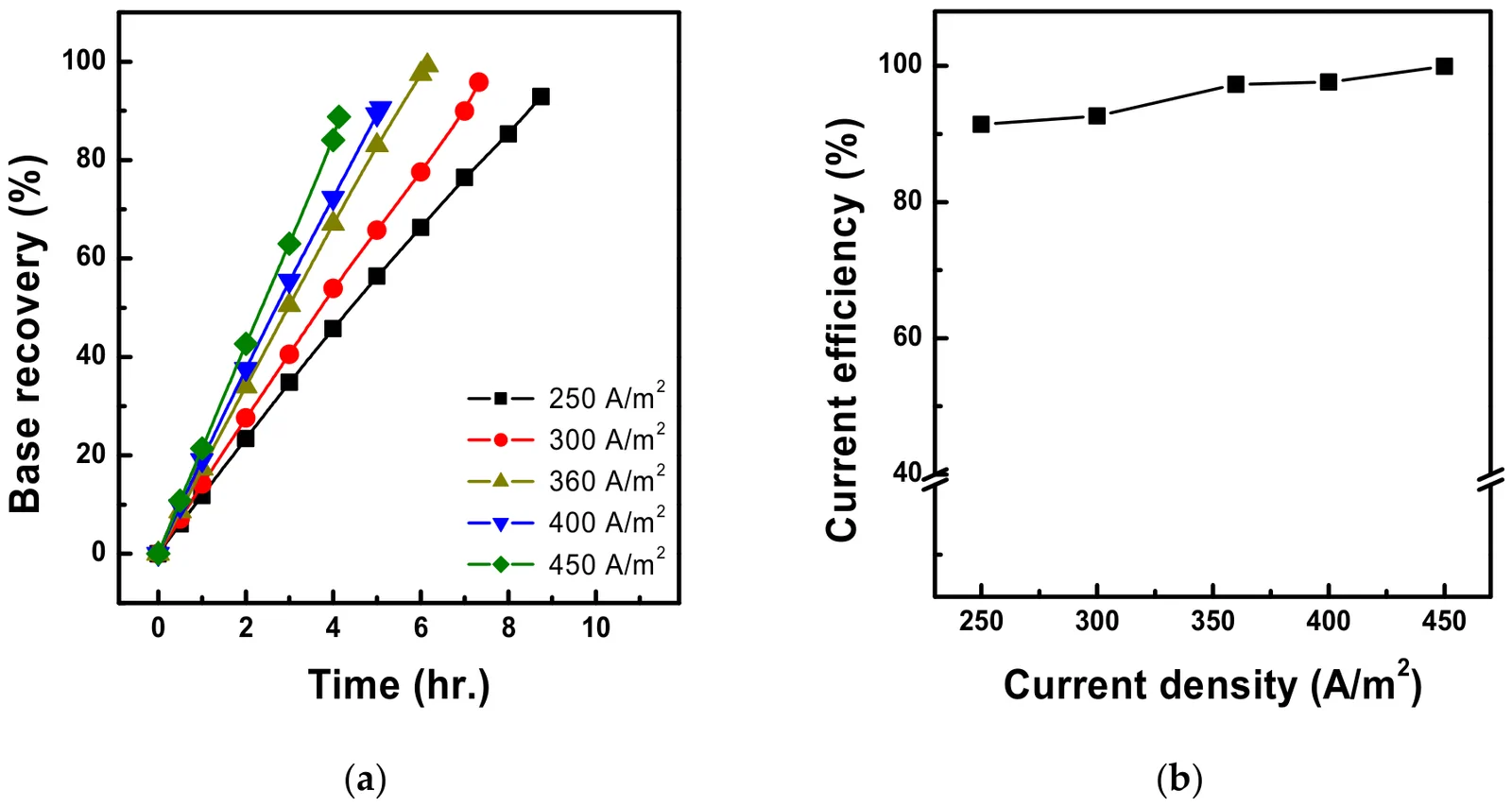
Effect of the current density (A/m^2^) on (**a**) NaOH recovery and (**b**) current efficiency (1.00 M Na_3_Cit (Feed), 0.1 M NaOH, and F/B = 1.5/1.5 (L), 30 °C).

As shown in Figure 4a,b, the base recovery and current efficiency showed clear differences depending on the current density. A high recovery of 95% or more was maintained in the 300–360 A/m^2^ range, with the highest value of 99.20% observed at 360 A/m^2^. In the low current density region of 250 A/m^2^, the recovery was relatively low at 92.85%, which is attributable to the slow net transport of Na^+^ due to the low electrochemical driving force and the accumulation of current loss from back-diffusion and recombination during long-term operation. A study by Gao et al. supports this result, reporting that operating time increased significantly while conversion efficiency decreased under low current density (10 mA/cm^2^) conditions [23]. Conversely, in the high current region of 400–450 A/m^2^, the recovery decreased to below 90%. This is attributed to the loss of excess OH^−^ through back-diffusion toward the feed solution chamber or co-ion leakage as the OH^−^ generation rate in the BM exceeds the Na^+^ supply rate through the CEM. A similar phenomenon has been reported in a previous study on a LiCl-LiOH system [24].

The current efficiency was lowest at 91.41% under the 250 A/m^2^ condition and highest at 97.27% at 360 A/m^2^. This is because the supply rate of Na^+^ through the CEM and the generation rate of OH^−^ in the BM reached dynamic equilibrium under these conditions, resulting in most of the applied current contributing to the generation of NaOH. Furthermore, at 450 A/m^2^, the current efficiency was high at 99.90%, but the recovery (88.74%) and the final NaOH concentration decreased, which was not consistent with actual process performance. This indicates an overestimation of current efficiency due to the limited range of concentration change caused by the short operating time (248 min). The energy consumption did not show a significant difference depending on current density but was lowest at 0.62 kWh/kg at 250 A/m^2^.

In summary, the 360 A/m^2^ condition exhibited the best performance in terms of base recovery and current efficiency. However, in the 360–450 A/m^2^ range, the cell voltage repeatedly exceeded 25 V for 5–10 min after the start of the process, and this high-voltage load could accelerate chemical and electrical degradation of the ion-exchange membrane during long-term repetitive operation, leading to a decrease in membrane lifetime. Therefore, based on a comprehensive consideration of process performance and long-term operational stability, the 300 A/m^2^ condition is determined to be the most desirable operating condition.

### 3.3. Effect of Initial Base Concentration

To evaluate the effect of initial base concentration on the BMED process, experiments were performed by varying the initial NaOH concentration in the base recovery chamber from 0.1 to 1.0 M. The experiments were conducted at a current density of 300 A/m^2^ using 1.0 M Na_3_Cit as the feed solution; in particular, 1.5 L each of the feed solution and the initial base solution were charged.

**Figure 5:**
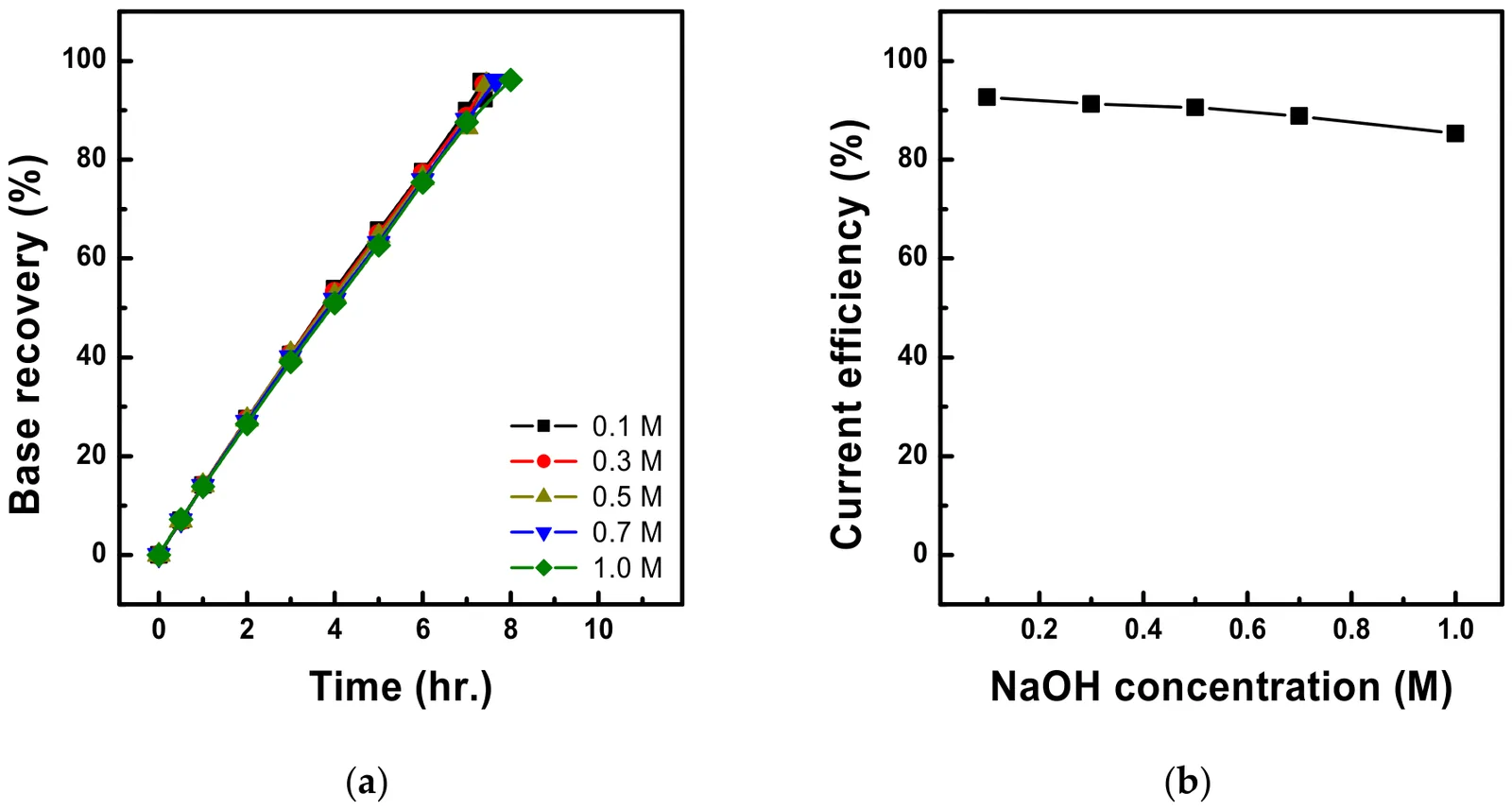
Effect of NaOH concentration (M) of initial base solution on (**a**) NaOH recovery and (**b**) current efficiency at 300 A/m^2^ (1.00 M Na_3_Cit (Feed), F/B = 1.5/1.5 (L), 30 °C).

As shown in Figure 5a, the base recovery was maintained at approximately 95% under all conditions and did not show a significant difference with changes in the initial base concentration. Conversely, as shown in Table 5, the final concentration of recovered NaOH increased markedly with increasing initial base concentration, with the lowest value at 2.41 M for 0.1 M and the highest at 2.99 M for 1.0 M. This suggests that initial base concentration is a more important factor than recovery in determining the final recovered concentration. The concentration of H_3_Cit formed as a complex salt in the feed solution chamber also increased slightly from 1.20 M to 1.31 M as the initial base concentration increased. As shown in Figure 5b, the current efficiency continuously decreased from 92.66% at 0.1 M to 85.27% at 1.0 M as the initial base concentration increased. Under high-concentration base conditions, as the OH^−^ concentration in the base recovery chamber increased, the concentration gradient with the feed solution chamber increased significantly, promoting the back-diffusion of OH^−^. Furthermore, in a high ionic strength environment, the selectivity of the CEM decreased, increasing co-ion leakage. These two mechanisms are considered to be responsible for the decrease in current efficiency.

The process time for different initial base concentrations ranged from 440 to 480 min, with no significant difference between conditions; the average voltage for each condition was also similar at around 19.07 V. This is likely because changes in initial NaOH concentration exerted a limited effect on the voltage rise rate and electrolyte composition. The water migration in the base recovery chamber was 23.63% at 0.1 M but increased to 28.97% at 1.0 M. In previous experiments, operating time had the greatest effect on water migration, but in this experiment, the drag effect from the osmotic pressure gradient due to increased initial base concentration was more dominant. An increase in water migration could cause total volume changes and affect downstream concentrations; however, the water migration (23–29%) observed in this experiment was at a level that did not cause significant problems for membrane stability or process operation. The energy consumption gradually increased from 0.64 to 0.70 kWh/kg as the initial base concentration increased.

The comprehensive experimental results confirmed that the initial base concentration is a more influential factor on the final recovered base concentration than the base recovery. Although recovering high-concentration NaOH under high-concentration conditions is advantageous, it results in decreased current efficiency and increased energy consumption. Therefore, to balance process efficiency and recovery concentration, operating in the 0.1 to 0.5 M range is most suitable.

### 3.4. Effect of Initial Base Volume

The effect of increasing the concentration of recovered NaOH was investigated by varying the volume of NaOH charged into the initial base recovery chamber from 0.75 to 2.00 L. The experiment was conducted at a current density of 300 A/m^2^; the feed solution chamber was charged with 1.5 L of 1.0 M Na_3_Cit, and the base recovery chamber was charged with 0.1 M NaOH, adjusted for each volume condition.

**Figure 6:**
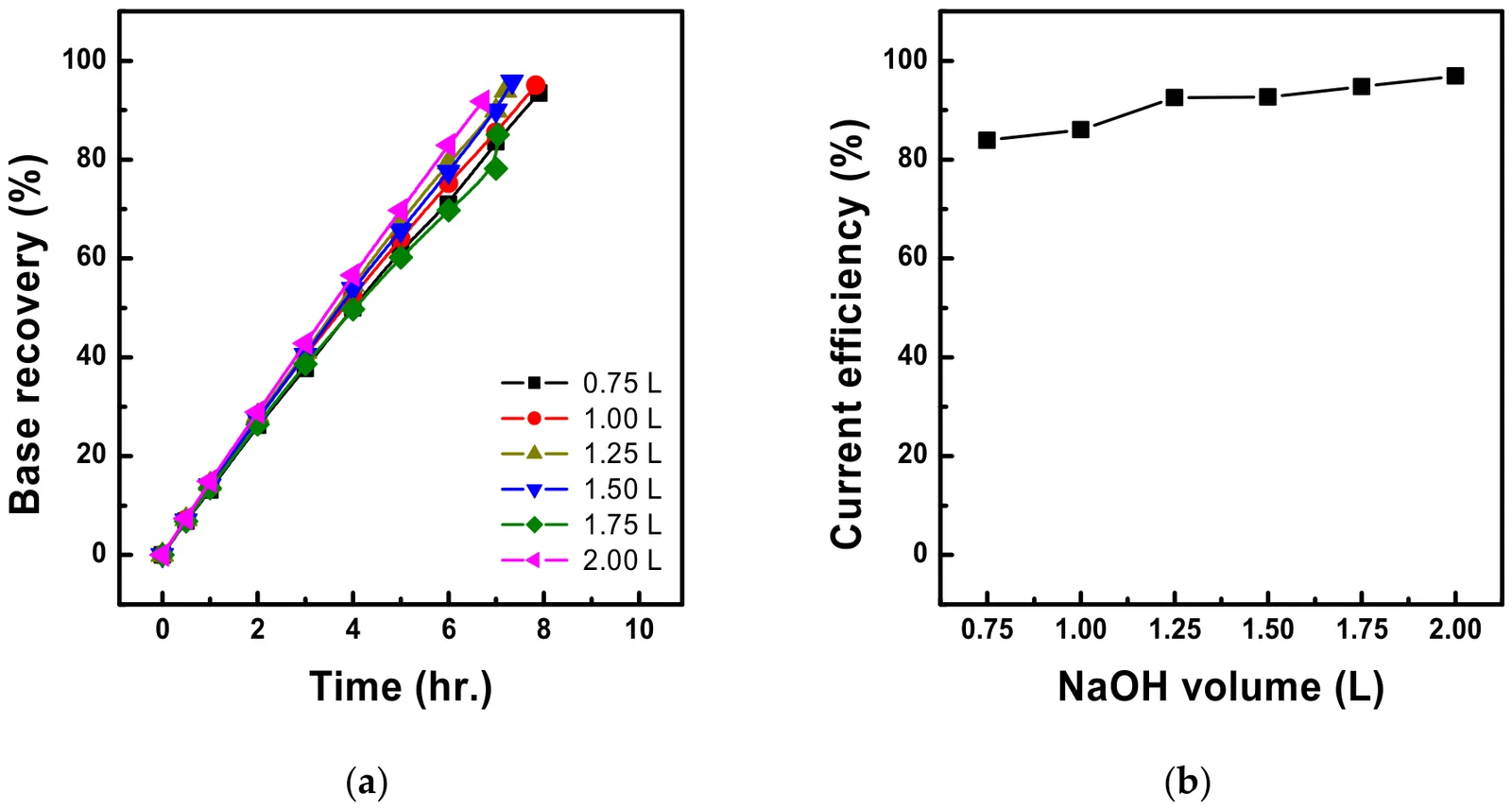
Effect of NaOH volume (L) of initial base solution on (**a**) NaOH recovery and (**b**) current efficiency at 300 A/m^2^ (1.00 M Na_3_Cit (Feed), 1.5 L; 0.1 M NaOH; and 30 °C).

As shown in Figure 6a, the base recovery was generally maintained in the range of 91–95% and did not show a significant difference, with the highest value of 95.78% at 1.50 L. Under low-volume conditions (0.75–1.00 L), a slight decrease in recovery was observed due to high water migration, while under high-volume conditions (2.00 L), the decrease in recovery was due to reduced coupling efficiency between OH^−^ generation and Na^+^ transport caused by the electrolyte dilution effect. Meanwhile, the final concentration of recovered NaOH showed a clear difference depending on the initial volume. The lower the initial volume, the higher the concentration at which NaOH was recovered, with the highest value of 3.56 M at 0.75 L and the lowest value of 1.88 M at 2.00 L. The concentration of H_3_Cit produced in the feed solution chamber also tended to increase as the initial base volume decreased, which is attributed to the more rapid concentration of produced citric acid as the feed solution volume decreases due to high water migration. From an industrial perspective, as the concentration of recovered NaOH directly affects the economics of downstream processes such as reintroduction and concentration, an excessively large initial volume is inappropriate because it can increase downstream concentration costs due to the dilution effect. As shown in Figure 6b, the current efficiency gradually increased with an increase in the initial base volume. Under low-initial-volume conditions, NaOH in the base recovery chamber was rapidly concentrated, and the OH^−^ concentration increased sharply, increasing the concentration gradient across the membrane and promoting the back-diffusion of OH^−^ and co-ion leakage. As a result, the effective charge contributing to NaOH generation decreases, lowering current efficiency. This is consistent with a previous study reporting that back diffusion and co-ion leakage are the main causes of decreased current efficiency with increasing acid-base concentration in the BMED process [25].

As seen in Table 6, the operating time gradually decreased as the initial volume increased, which is because as the initial volume increases, rapid fluctuations in concentration and electrolyte composition are mitigated, allowing the voltage increase rate to be stably maintained. Based on the base recovery chamber, the water migration markedly increased from 15.14% under the 2.00 L condition to 60.09% under the 0.75 L condition as the initial volume decreased. Under low-initial-volume conditions, the OH^−^ concentration in the base recovery chamber increased rapidly in a short time, significantly increasing the osmotic pressure gradient across the membrane. Electro-osmotic drag is considered to have increased considerably as a result, contributing to the high water migration observed. The water migration in the feed solution chamber also gradually decreased from 30.05% under the 0.75 L condition to 20.18% under the 2.00 L condition. Moreover, the volume loss of the feed solution was reduced.

In summary, although recovering high-concentration NaOH with a decreasing initial base volume is advantageous, it is accompanied by a decrease in current efficiency and an increase in energy consumption, thereby limiting process efficiency. Therefore, it is desirable to select appropriate initial volume conditions by balancing the recovered concentration and process efficiency.

### 3.5. Optimal Process Design

To determine the optimal operating conditions for a two-compartment BMED process to recover citric acid and NaOH from a sodium citrate (Na_3_Cit) solution, water migration, operating time, base recovery and recovered concentration, current efficiency, and energy consumption were comprehensively evaluated. The main operating parameters were the feed solution concentration, current density, and the initial base concentration and volume.

For the feed solution concentration, excessively low concentrations led to reduced recovery, which is considered to be attributable to intensified concentration polarization from insufficient ion supply. Conversely, excessively high concentrations caused decreases in recovery and current efficiency because of increased solution viscosity and intensified concentration polarization. After comprehensive consideration of overall performance indicators, a 1.0 M Na_3_Cit concentration was identified as the most stable and balanced operating condition in terms of recovery, current efficiency, and energy consumption. Regarding current density, a comprehensive evaluation of recovery and current efficiency showed that 360 A/m^2^ provided the best performance. However, under these conditions, the cell voltage repeatedly exceeded 25 V for approximately 5 to 10 min immediately after startup. This high-voltage load may accelerate chemical and electrical degradation of the ion-exchange membrane during long-term operation, reducing membrane lifetime. Therefore, considering both process performance and long-term operational stability, 300 A/m^2^ is determined to be the most desirable operating condition. The concentration and volume of the initial base (NaOH) are process parameters that must be adjusted according to the process objective. The final concentration of recovered NaOH increased with a higher initial base concentration, while current efficiency showed a clear downward trend. Considering this result, it is appropriate to operate with an initial base concentration in the 0.1–0.5 M range. For the initial base volume, high-concentration NaOH can be recovered by reducing the volume, but excessive reduction is not desirable because it decreases current efficiency and increases energy consumption.

**Figure 7:**
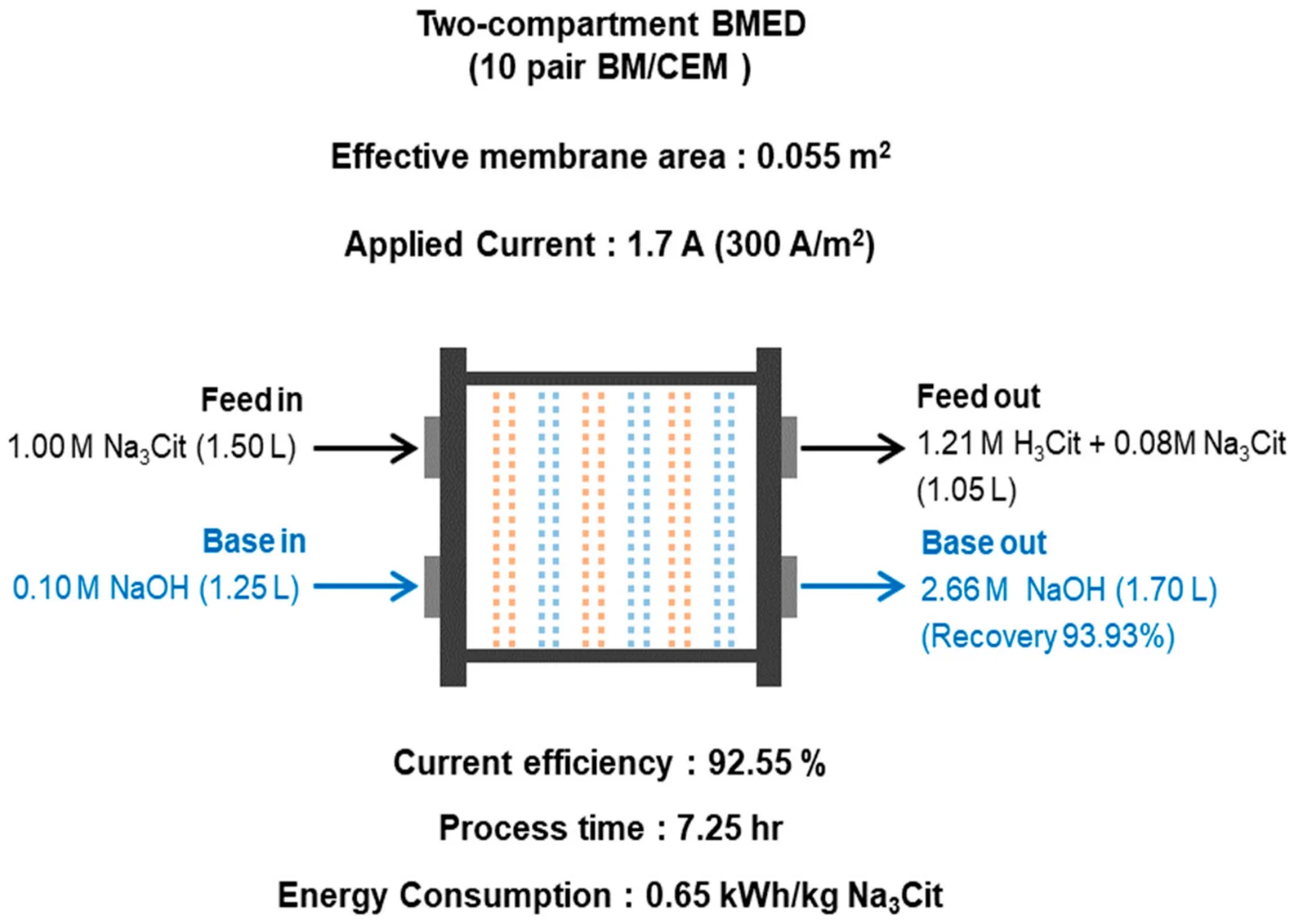
Schematic of the proposed process (300 A/m^2^, initial concentration of feed: 1.00 M Na_3_Cit, initial concentration of base: 0.1 M, F/B = 1.50/1.25 (L), and 30 °C). The applied current density and current were calculated based on the area of a single membrane pair (0.0055 m^2^).

The optimal operating conditions derived from these results are presented in Figure 7. The optimal conditions were set as follows: a feed solution concentration of 1.0 M Na_3_Cit, a current density of 300 A/m^2^, an initial base concentration of 0.1 M NaOH, and an initial base volume of 1.25 L. Under these conditions, NaOH recovery was 93.93%, and the concentration of recovered NaOH was 2.66 M. In addition, the current efficiency was stably maintained at 92.55%, and energy consumption was relatively low at 0.65 kWh/kg, making the process efficient. Although a high-purity caustic soda solution can be recovered in the base chamber, the citric acid recovered from the feed solution chamber is mixed with residual citrate. Under the optimal condition, the residual Na^+^ concentration in the feed chamber, measured via ICP-OES, corresponded to 0.270 mol of unreacted Na_3_Cit, indicating that the recovered acid consisted of approximately 94.00 mol% H_3_Cit and 6.00 mol% residual Na_3_Cit. Whether this Na_3_Cit-containing citric acid mixture can be directly reused in the leaching process without further purification requires additional experimental verification. To assess the reproducibility of the process performance and the stability of the membrane stack under repeated use, the experiment under the optimal condition was independently repeated three times over the course of the study. The NaOH recovery, current efficiency, and energy consumption varied by less than 3% across these repeated trials, indicating that the process performance was reproducible and that the membrane stack, which was reused with intermediate washing throughout the entire study, did not undergo significant performance degradation over repeated and extended use.

## 4. Conclusions

In this study, the effects of major process parameters on the effective separation and recovery of citric acid and caustic soda (NaOH) from citrate wastewater (Na_3_Cit) using a two-compartment bipolar membrane electrodialysis (BMED) system were investigated. The following conclusions were obtained:

(1) At low feed concentrations, recovery decreased, which can be attributed to increased membrane resistance; at high concentrations, both current efficiency and recovery decreased, likely reflecting increased viscosity and concentration polarization. Collectively, 1.0 M Na_3_Cit was identified as the most stable operating concentration.
(2) Current density exerted the greatest influence on operating time. While operating time was shortened with increased current density, excessively high current densities led to lower base recovery and repeated high-voltage periods during initial operation. Therefore, considering both process performance and long-term operational stability, a current density of 300 A/m^2^ is most desirable.
(3) Increasing the initial base concentration raised the final NaOH concentration but decreased current efficiency, likely due to OH^−^ back-diffusion and co-ion leakage; operating in the 0.1–0.5 M range best balanced efficiency and recovered concentration.
(4) A smaller initial base volume enabled recovery of more concentrated NaOH but at the cost of lower current efficiency and higher energy consumption, requiring a trade-off between concentration and process efficiency.

Based on these results, the optimal operating conditions were determined to be 1.00 M Na_3_Cit, 300 A/m^2^, an initial base concentration of 0.1 M, and an initial base volume of 1.25 L, achieving a NaOH recovery of 93.93%, current efficiency of 92.55%, and energy consumption of 0.65 kWh/kg. This study experimentally demonstrated that Na_3_Cit wastewater generated from a citric acid-based leaching process can be treated via BMED using an environmentally friendly method, recovering high-purity NaOH and citric acid containing residual unreacted Na_3_Cit and confirming that a two-compartment BMED process has practical potential as a resource-circulating treatment technology for citrate wastewater. However, this study was conducted under lab-scale batch operating conditions. For actual process application, further research is required on the transition to a continuous process, evaluation of membrane stability under long-term repeated operation, and scale-up.

## Figures and Tables

**Table 1:** Specifications of manufactured equipment (bipolar membrane electrodialyzer).

Manufactured Equipment	
Effective membrane area	Total: 0.055 m^2^ (10 membrane pairs); 0.0055 m^2^ per pair
Operating mode	Constant voltage and Constant current
Terminating mode	Voltage, Current, and Conductivity
Terminating condition	Current: 0.00–6.00 A Conductivity: 0.0–200 mS/cm
Line capacity	150 mL
Standard processing capacity	-
Upper temperature limit	40 °C
Upper chamber capacity limit	2.5 L

**Table 2:** Specifications of BM and CEM.

Membrane	BM (Neosepta BPU)	CEM (Neosepta CMB)
Type	-	Strong acid (Na type)
characteristics	Water splitting ∙ Voltage 1.0 V ∙ Efficiency ≥ 0.98	High mechanical strength/alkali resistance
Electric resistance ($\Omega •{cm}^{2}$)	-	4.5
Burst strength (MPa)	≥0.70	≥0.40
Thickness (mm)	0.28	0.21
Application	∙ BMED	∙ Alkali recovery ∙ BMED ∙ Diaphragm
Temperature (°C)	≤40	≤60
pH	-	0–14

**Table 3:** Effects of initial concentration of the feed solution on time and water migration, NaOH recovery, acid in the mixed solution, average voltage, and energy consumption at 360 A/m^2^ (0.1 M NaOH, F/B = 1.5/1.5(L), 30 °C).

Current Density (A/m^2^)	Initial Feed Sol. (M)	Time (min)	Water Migration to Base (%)	Recovery of NaOH (%) (M)	Acid in Mixed Sol. (M)	Average Voltage (V)	Energy Consumption (kWh/kg) *
360	0.30	76	5.66	72.83 (0.72)	0.23	23.74	0.71
	0.50	162	13.12	89.44 (1.27)	0.68	22.02	0.69
	0.75	244	16.65	89.39 (1.81)	1.00	21.17	0.59
	1.00	369	22.07	99.20 (2.52)	1.45	21.01	0.67
	1.25	447	25.15	92.45 (2.85)	1.52	20.73	0.69
	1.50	562	28.96	92.10 (3.30)	2.02	20.40	0.71

* Energy consumption to process 1 kg of sodium citrate.

**Table 4:** Effects of current density on process time, water migration, NaOH recovery, acid in the mixed solution, average voltage, and energy consumption (1.00 M Na_3_Cit (Feed), 0.1 M NaOH, F/B = 1.5/1.5 (L), and 30 °C).

Initial Feed Sol. (M)	Current Density (A/m^2^)	Time (min)	Water Migration to Base (%)	Recovery of NaOH (%) (M)	Acid in Mixed Sol. (M)	Average Voltage (V)	Energy Consumption (kWh/kg) *
1.00	250	525	23.63	92.85 (2.33)	1.21	18.13	0.62
	300	440	23.63	95.78 (2.41)	1.20	19.08	0.64
	360	369	22.07	99.20 (2.52)	1.28	21.01	0.67
	400	305	21.93	90.55 (2.31)	1.14	21.55	0.69
	450	248	20.10	88.74 (2.30)	1.10	22.59	0.68

* Energy consumption to process 1 kg of sodium citrate.

**Table 5:** Effects of NaOH concentration (M) of initial base solution on process time, water migration, NaOH recovery, acid in the mixed solution, average voltage, and energy consumption at 300 A/m^2^ (1.00 M Na_3_Cit (Feed), F/B = 1.5/1.5 (L), and 30 °C).

Initial Feed Sol. (M)	Initial Base Sol. (M)	Time (min)	Water Migration to Base (%)	Recovery of NaOH (%) (M)	Acid in Mixed Sol. (M)	Average Voltage (V)	Energy Consumption (kWh/kg) *
1.00	0.1	440	23.63	95.78 (2.41)	1.20	19.08	0.64
	0.3	444	24.19	95.23 (2.53)	1.23	19.07	0.65
	0.5	447	25.72	95.14 (2.66)	1.27	19.08	0.66
	0.7	460	27.36	95.99 (2.80)	1.29	19.06	0.67
	1.0	480	28.97	96.15 (2.99)	1.31	19.05	0.70

* Energy consumption to process 1 kg of sodium citrate.

**Table 6:** Effects of NaOH volume (L) of initial base solution on time and water migration, recovery of base, acid in mixed solution, average voltage, and energy consumption at 300 A/m^2^ (1.00 M Na_3_Cit (Feed), 1.5 L; 0.1 M NaOH; and 30 °C).

Initial Base Sol. (L)	Time (min)	Water Migration (%)		Recovery of NaOH (%) (M)	Acid in Mixed Sol. (M)	Average Voltage (V)	Energy Consumption (kWh/kg) *
		Base	Feed **				
0.75	474	60.09	−30.05	93.40 (3.56)	1.28	83.88	0.71
1.00	470	41.70	−27.80	95.03 (3.09)	1.25	86.07	0.71
1.25	432	29.72	−24.76	93.93 (2.66)	1.21	92.55	0.65
1.50	440	23.63	−23.63	95.78 (2.41)	1.20	92.66	0.64
1.75	422	19.38	−21.91	93.77 (2.15)	1.16	94.76	0.63
2.00	403	15.14	−20.18	91.76 (1.88)	1.12	96.92	0.62

* Energy consumption to process 1 kg of sodium citrate. ** A negative value indicates that the feed chamber volume decreased as a result of water migration toward the base chamber.

## Data Availability

All data generated or analyzed during this study are included in this published article.
